# A global long-term ocean surface daily/0.05° net radiation product from 1983–2020

**DOI:** 10.1038/s41597-022-01419-x

**Published:** 2022-06-14

**Authors:** Hui Liang, Bo Jiang, Shunlin Liang, Jianghai Peng, Shaopeng Li, Jiakun Han, Xiuwan Yin, Jie Cheng, Kun Jia, Qiang Liu, Yunjun Yao, Xiang Zhao, Xiaotong Zhang

**Affiliations:** 1 The State Key Laboratory of Remote Sensing Science, Jointly Sponsored by Beijing Normal University and Institute of Remote Sensing and Digital Earth of Chinese Academy of Sciences, Beijing, 100875 China; 2 China and Beijing Engineering Research Center for Global Land Remote Sensing Products, Institute of Remote Sensing Science and Engineering, Faculty of Geographical Science, Beijing Normal University, Beijing, 100875 China; 3 Department of Geographical Sciences, University of Maryland, College Park, MD 20742 USA; 4 College of Global Change and Earth System Science, Beijing Normal University, Beijing, 100875 China

**Keywords:** Physical oceanography, Physical oceanography

## Abstract

The all-wave net radiation (*R*_*n*_) on the ocean surface characterizes the available radiative energy balance and is important to understand the Earth’s climate system. Considering the shortcomings of available ocean surface *R*_*n*_ datasets (e.g., coarse spatial resolutions, discrepancy in accuracy, inconsistency, and short duration), a new long-term global daily *R*_*n*_ product at a spatial resolution of 0.05° from 1983 to 2020, as part of the Global High Resolution Ocean Surface Energy (GHOSE) products suite, was generated in this study by fusing several existing datasets including satellite and reanalysis products based on the comprehensive *in situ* measurements from 68 globally distributed moored buoy sites. Evaluation against *in-situ* measurements shows the root mean square difference, mean bias error and correlation coefficient squared of 23.56 Wm^−2^, 0.88 Wm^−2^ and 0.878. The global average ocean surface *R*_*n*_ over 1983–2020 is estimated to be 119.71 ± 2.78 Wm^−2^ with a significant increasing rate of 0.16 Wm^−2^ per year. GHOSE *R*_*n*_ product can be valuable for oceanic and climatic studies.

## Abstract (summary)

**Table:** 

Measurement(s)	The all-wave net radiation
Technology Type(s)	fusion method

## Background & Summary

The all-wave net radiation (*R*_*n*_) at the ocean surface, which characterizes the available radiative energy balance on the ocean surface, is the difference between radiation from the sun and the atmosphere, and that emitted and reflected from the ocean surface^1^. The ocean surface *R*_*n*_ is mathematically represented as the sum of the net shortwave radiation (*R*_*ns*_) and the net longwave radiation (*R*_*nl*_) on the ocean surface, as shown in Eq. (1):

1 $$\begin{array}{lll}{{\boldsymbol{R}}}_{{\boldsymbol{n}}} & = & {{\boldsymbol{R}}}_{{\boldsymbol{ns}}}+{{\boldsymbol{R}}}_{{\boldsymbol{nl}}}\\ {{\boldsymbol{R}}}_{{\boldsymbol{ns}}} & = & {{\boldsymbol{R}}}_{{\boldsymbol{si}}}-{{\boldsymbol{R}}}_{{\boldsymbol{so}}}\\ {{\boldsymbol{R}}}_{{\boldsymbol{nl}}} & = & {{\boldsymbol{R}}}_{{\boldsymbol{li}}}-{{\boldsymbol{R}}}_{{\boldsymbol{lo}}}\end{array}$$

where *R*_*si*_ is the incoming shortwave radiation (negative upward, Wm^−2^), *R*_*so*_ is the reflected outgoing shortwave radiation (Wm^−2^), *R*_*li*_ is the incoming longwave radiation (Wm^−2^), and *R*_*lo*_ is the outgoing longwave radiation (Wm^−2^).

The ocean is the largest body of heat storage in the Earth’s climate system because of its large specific heat capacity in seawater and high occupancy of the Earth’s surface area (71%). Ocean heat flux, which is composed of the ocean surface radiative heat flux (*R*_*n*_) and turbulent heat flux (latent heat flux and sensible heat flux)^2^, can regulate the heat balance in the Earth’s system through frequent air-sea energy exchange and by transferring the heat into the underlying body of water and the overlying air^3^. The ocean heat flux is closely related to the Earth’s Energy Imbalance (EEI)^4^, which is represented by the difference between the incident solar radiation and the outgoing longwave radiation of the Earth, and is thought to characterize the planet’s climate and global warming caused by human activities^5^. Hence, as it is a major component of ocean heat flux, variations in the ocean surface *R*_*n*_ can directly affect it and, in turn, influence atmospheric and oceanic circulations, and even climate change at different spatio-temporal scales^1,6,7^. In addition, the ocean surface *R*_*n*_ plays an important role in the oceanic and climate-related research as an essential input to most models^8^. Therefore, an accurate estimation of the ocean surface *R*_*n*_ is important for various applications and understanding the state of the Earth’s climate system.

However, *R*_*n*_ or the four radiative components (see Eq. (1)) are not routinely measured at the ocean surface, and the ocean surface *R*_*n*_ is usually estimated by several parameterizations or empirical formulas, or is obtained from various products by summing their radiative components. And almost all available products have coarse spatial resolutions, only with the finest spatial resolution of 0.25°. Moreover, some products cover only the ice-free oceans (e.g., the Japanese Ocean Flux Data Sets with Use of Remote Sensing Observations, version3 <J-OFURO3>^2^, the Objectively Analyzed Air-Sea Flux <OAFlux>^9,10^, and TropFlux^11^), and the uncertainty in the *R*_*n*_ over the coastal seas is large for most products owing to their coarse spatial resolutions^12^. Furthermore, other problems in these products, such as the discrepancy in accuracy, inconsistency^13^, and short duration, also limit the wide use of the ocean surface *R*_*n*_ from them. Therefore, a reliable, long-term time series of the ocean surface *R*_*n*_ dataset with a high accuracy and high spatio-temporal resolution is needed.

Data fusion, as an effective means of improving parameter estimation, has been applied widely, and works well as long as a sufficient number of datasets are available. It can combine estimates from multiple algorithms or high-level products to obtain new and better estimates, which are highly accurate and have high spatio-temporal resolution, through statistical methods^14,15^, including classic methods (e.g., regression, Bayesian Model Averaging (BMA)^16,17^, Simple Taylor Skill (STS)^18^, Empirical Orthogonal Function (EOF)^19^), and various machine learning methods (e.g., Multi-resolution Tree (MRT)^20^, Supporting Vector Machine (SVM)^6^, and artificial neural networks (ANN)^21^). A large number of datasets with various parameters has been successfully generated by data fusion, such as those for the land surface *R*_*nl*_^22^, terrestrial latent heat flux^23^, the surface radiation budget (SRB) over the Tibetan Plateau^24^, land surface broadband emissivity^25^, Leaf Area Index (LAI)^19^, and surface broadband albedo^26^.Overall, data fusion is a very useful technique for improving product quality regardless of the parameter at hand. Therefore, By considering a plenty of available ocean surface *R*_*n*_ datasets, data fusion was applied for generating a new better one.

In this study, after exploring four commonly used data fusion methods including stepwise regression (SR), STS, BMA and back-propagation neural network (BP) thoroughly, a new daily ocean surface *R*_*n*_ dataset at a spatial resolution of 0.05° from 1983 to 2020, which was included in the Global High resolution Ocean Surface Energy (GHOSE) data suite, was generated with the best forming SR method. Afterwards, GHOSE daily *R*_*n*_ was evaluated comprehensively.

## Methods

### Calculation of the *in situ* ocean surface *R*_*n*_

**Fig. 1:**
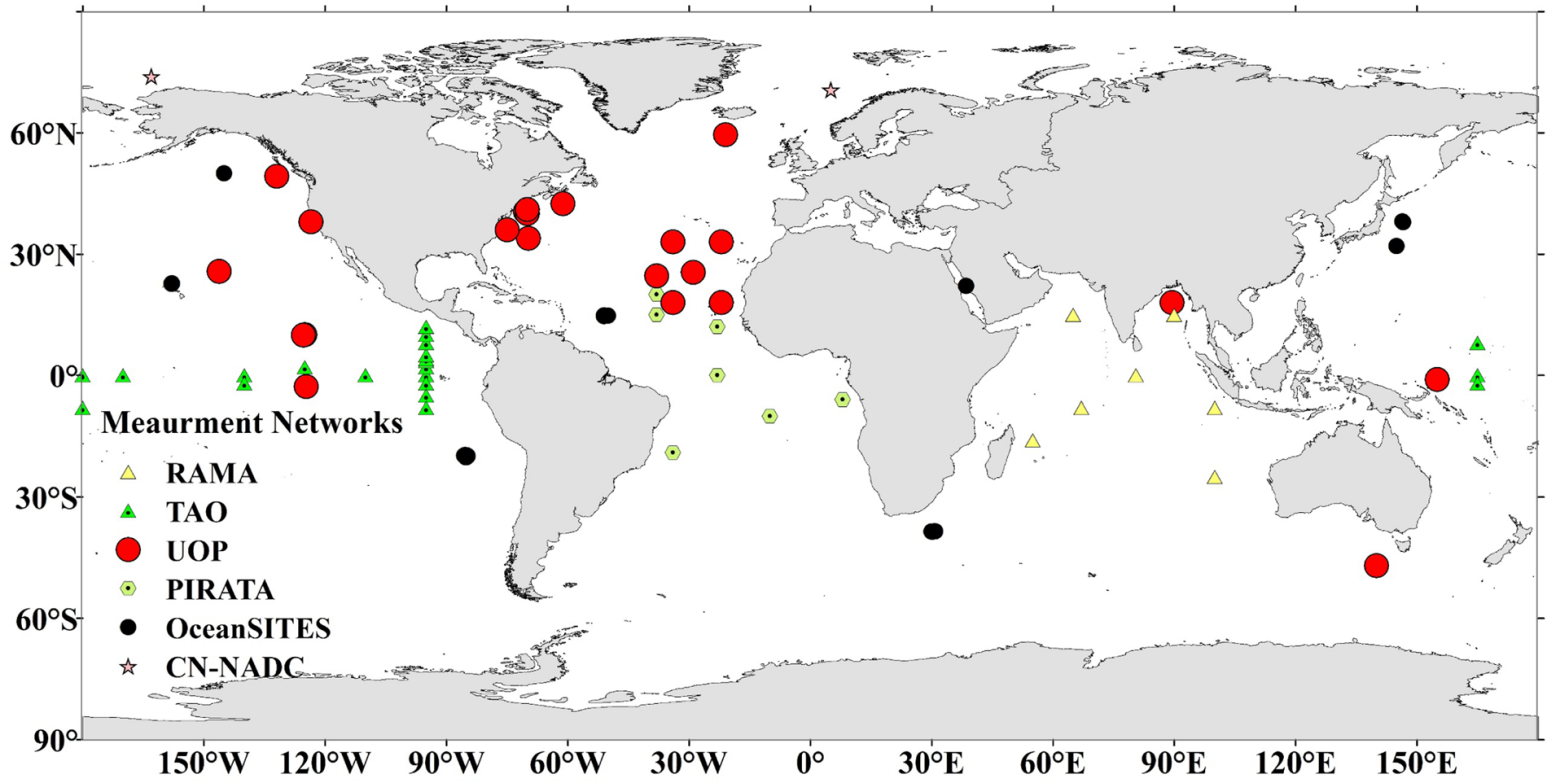
The distribution of the 68 moored buoys from six observation networks.

In this study, 68 moored buoy sites from six observation networks were collected from 1988 to 2019. The spatial distribution of the buoy sites is shown in Fig. 1. They were located in global oceans, and were mainly distributed over seas at mid-to-low latitudes, especially near the Equator. However, few buoys were located in seas at high latitudes (>50°), especially in the Southern Hemisphere.

Table 1 lists detailed information on the six observation networks to which the 68 moored buoy sites belonged, including the Pilot Research Moored Array in the Tropical Atlantic (PIRATA), the Tropical Atmosphere Ocean (TAO), the Research Moored Array for African-Asian-Australian Monsoon (RAMA), the Ocean Sustained Interdisciplinary Timeseries Environment Observation System (OceanSITES), the Upper Ocean Processes Group (UOP), and the Chinese National Arctic and Antarctic Center (CN-NADC). Among them, PIRATA, TAO, and RAMA are the major components of the Global Tropical Moored Buoy Array (GTMBA) Program, whose target of measurements are the tropical seas, including the Atlantic^27^, Pacific^28^, and Indian Oceans^29^. Their measurements are highly accurate owing to strict quality control procedures^30^. OceanSITES is a worldwide system for collecting and providing data at fixed locations in the open ocean^31^. The UOP network offers data on the upper ocean and at the air–sea interface in the north Atlantic. Measurements of three parameters (*R*_*si*_, *R*_*li*_, and the sea surface temperature <SST>) provided by 66 sites from the above five networks were used to calculate *R*_*n*_. Specifically at the moored buoys, two radiative measurements were taken directly using the Eppley Laboratory’s precision spectral pyranometer (PSP) and the precision infrared radiometer (PIR)^32^. The SST was the adjusted sea bulk temperature measured 2 m under the sea surface using Sea Bird Electronics (SBE37/39), and adding −0.17 K to the result when the wind speed exceeded 4 ms^−1 ^^33–35^. Unlike the other 66 moored buoy sites, the two moored sites from CN-NADC, located in the North Polar seas, could measure four radiative components, but their periods of observation were short (five days in 1999 at CNNADC_NP site, and 158 days from 2012 to 2014 at CNNADC_NW_sea site). The measurements provided by the six networks have been widely used, such as on research of climate variation^36,37^, heat budget^38,39^, and air-sea radiation fluxes^40^. Even still, the high quality measurements identified by their publishers^41^ were manually checked, and the final selected measurements were used. All the selected *in situ* measurements were transferred into the Coordinated Universal Time (UTC) format, and the daily means were then calculated as long as all measurements were available for a given day.

**Table 1:** The information of global six observing networks.

Network	No. of sites	Variables measuring	Observation frequency	Site URL
OceanSITES	8	*R*_*si*_*, R*_*li*_*, SST*	1 hour	http://www.oceansites.org/ (last accessed: 11 Feb. 2021)
TAO	21	*R*_*si*_*, R*_*li*_*, SST*	10 min	https://www.pmel.noaa.gov/gtmba/ (last accessed: 21 Apr. 2022)
RAMA	7	*R*_*si*_*, R*_*li*_*, SST*	10 min	https://www.pmel.noaa.gov/gtmba/ (last accessed: 21 Apr. 2022)
PIRATA	7	*R*_*si*_*, R*_*li*_*, SST*	10 min	https://www.pmel.noaa.gov/gtmba/ (last accessed: 21 Feb. 2022)
UOP	23	*R*_*si*_*, R*_*li*_*, SST*	1 hour	http://uop.whoi.edu/index.html (last accessed: 11 Feb. 2021)
CN-NADC	2	*R*_*si*_*, R*_*li*_*, R*_*so*_*, R*_*lo*_	1 hour	http://www.chinare.org.cn (last accessed: 11 Feb. 2021)

As mentioned above, neither *R*_*n*_ nor the four radiative components (see Eq. (1)) are routinely measured, but the downward radiation (*R*_*si*_ and *R*_*li*_) were measured instead at almost all moored buoys. Hence, the *in situ* ocean surface *R*_*n*_ at the moored buoys needed to be first calculated based on the moored measurements and ancillary information (*R*_*si*_, *R*_*li*_, and SST) in Eq. (2), except at the two sites from CN-NADC:

2 $$\begin{array}{lll}{{\boldsymbol{R}}}_{{\boldsymbol{n}}} & = & {{\boldsymbol{R}}}_{{\boldsymbol{ns}}}+{{\boldsymbol{R}}}_{{\boldsymbol{nl}}}\\ {{\boldsymbol{R}}}_{{\boldsymbol{ns}}} & = & {{\boldsymbol{R}}}_{{\boldsymbol{si}}}\times ({\bf{1}}-{{\boldsymbol{\alpha }}}_{{\boldsymbol{ocean}}})\\ {{\boldsymbol{R}}}_{{\boldsymbol{nl}}} & = & {{\boldsymbol{\varepsilon }}}_{{\boldsymbol{ocean}}}\times ({{\boldsymbol{R}}}_{{\boldsymbol{li}}}-{\boldsymbol{\delta }}\times {{\boldsymbol{SST}}}^{4})\end{array}$$

where *δ* is the Stefan-Boltzmann constant (5.6697 × 10^−8^ [W/(m^2^∙K^4^)]), *α*_*ocean*_ is the ocean surface broadband albedo, *ε*_*ocean*_ is the ocean surface broadband emissivity, and *α*_*ocean*_ and *ε*_*ocean*_ were obtained from the GHOSE Ocean Water Albedo (OWA) and the broadband emissivity (BBE) products, respectively. The GHOSE OWA was developed based on satellite reflectance data via the Moderate-Resolution Imaging Spectroradiometer (MODIS) and ancillary data from MERRA2, obtained by Feng *et al*.^42^ and Wang *et al*.^43^ by using a three-component reflectance model at a resolution of 10 km from 1982 to 2019. It performed well after being validated with other parameterization methods. The GHOSE BBE is the first global ocean surface emissivity product with a resolution of 10 km from 1981 to 2019, and was generated from the MERRA2 reanalysis product developed by Cheng *et al*.^44^, It considers the effects of wind speed and sea foam. The authors claimed that uncertainty in the daily *ε*_*ocean*_ was less than 0.003 under wind-free conditions^45^. *α*_*ocean*_ and *ε*_*ocean*_ were extracted directly according to the locations of the 66 moored buoys. According to Peng *et al*.^12^, the uncertainty in the calculated *in situ* daily ocean surface *R*_*n*_ could be tolerated even given uncertainties in the other parameters.

In summary, 69,923 samples of the *in situ* ocean surface *R*_*n*_ at the daily scale were calculated and used in this study. Specifically, 70% samples from 1988 to 2016 at each moored buoy were randomly selected for the fusion model development, and the other 30% samples were used for independent validation. Moreover, all samples in 2017–2019 (No. of samples = 4,734) were used for evaluation.

### Products for fusion

The products that can provide the ocean surface *R*_*n*_ can be roughly divided into four categories: remotely sensed products, reanalysis products, reconstructed products, and ship-based products. Remotely sensed products are usually based on satellite observations, such as the Clouds and Earth’s Radiant Energy Systems synoptic Edition4A^46^ (CERES SYN1deg_Ed4A, CERES4 hereinafter), the International Satellite Cloud Climatology Project radiative flux D-series^29^ and H-series^47,48^ (ISCCP-FD and ISCCP-FH), and Global Energy and Water Cycle Experiment - Surface Radiation Budget^49,50^ (GEWEX-SRB); reanalysis products are derived from a data assimilation system by merging the available observations with model simulations^51^, such as the fifth generation European Centre for Medium-Range Weather Forecasts (ECMWF) atmospheric Re-Analysis^52^ (ERA5) and its previous version ERA-Interim^7^, the Japanese 55-year Reanalysis^53^ (JRA-55), the Modern-Era Retrospective analysis for Research and Applications Version2^54^ (MERRA2), and the National Centers for Environmental Prediction Department Reanalysis2^55^ (NCEP R2). These two kinds of products have mostly global coverage with a relative coarse spatial resolution. Although the reanalysis products cover more years than remotely sensed products, the accuracy of the latter in terms of the radiative components is generally superior to that of most reanalysis products for land^56^ and ocean surfaces^57^. Reconstructed products are generated by combining different kinds of products (e.g., remotely sensed and reanalysis products) through interpolation or other methods^11^, such as the aforementioned TropFlux, OAFlux, and J-OFURO3, and their performance is majorly determined by the original products. Ship-based products are calculated by means of parameterizations by using *in situ* measurements and then gridded by interpolation^9^, such as NOCS Surface Flux Dataset v2.0^9^ (NOCS V2.0), hence, their accuracies heavily depend on the quality of observations, the performance of the parameterization methods, and the re-gridding methods.

Take characteristics of all products into account, values of the ocean surface *R*_*n*_ from 11 products were applied in this study. Specifically, six products covering 1988–2016 were used for fusion including two remotely sensed products (CERES4 and ISCCP-FH) and four reanalysis products (JRA-55, MERRA2, NCEP R2, and ERA5), and five products were used for comparison including one remotely sensed product (GEWEX-SRB), three reconstructed products (TropFlux, OAFlux, and J-OFURO3), and one ship-based product (NOCS V2.0). Note that J-OFURO3 which was thought to be one of the best products in ocean surface *R*_*n*_^13^ was mainly considered as a reference. Table 2 lists the characteristics of the 11 used ocean surface *R*_*n*_ products.

**Table 2:** The characteristics of the 11 used ocean surface *R*_*n*_ products.

Product	Spatial Resolution	Temporal Resolution	Period	Coverage	URL
CERES4	1°	1 hourly	2000-	Global	https://ceres.larc.nasa.gov (last accessed: 18 Jun. 2021)
ISCCP-FH	1°	3 hourly	1983–2017	Global	https://isccp.giss.nasa.gov/ (last accessed: 18 Jun. 2021)
GEWEX-SRB	1°	3 hourly	1983–2007	Global	http://www.gewex.org/ (last accessed: 18 Jun. 2021)
ERA5	31 km(T639)	hourly	1950-	Global	https://www.ecmwf.int/en/ (last accessed: 18 Jun. 2021)
JRA55	T319(~55 km)	3 hourly	1958-	Global	https://rda.ucar.edu/datasets/ds628.0/ (last accessed: 18 Jun. 2021)
MERRA2	1/2° × 2/3°	hourly	1979-	Global	https://gmao.gsfc.nasa.gov/reanalysis/MERRA-2/ (last accessed: 18 Jun. 2021)
NCEP R2	T62(200 km)	6 hourly	1979-	Global	https://rda.ucar.edu/datasets/ds091.0/ (last accessed: 18 Jun. 2021)
OAFlux	1°	daily	1985–2009	Global ice-free ocean	http://oaflux.whoi.edu/ (last accessed: 18 Jun. 2021)
TropFlux	1°	daily	1979-	30°S-30°N ocean	https://incois.gov.in/tropflux/ (last accessed: 18 Jun. 2021)
J-OFURO3	0.25°	daily	1988–2013	Global ice-free ocean	https://j-ofuro.scc.u-tokai.ac.jp/en (last accessed: 18 Jun. 2021)
NOCS v2.0	1°	monthly	1973–2014	Global ocean	http://www.noc.soton.ac.uk/ (last accessed: 18 Jun. 2021)

Since only radiative components could be provided by the 11 products, the ocean surface *R*_*n*_ should be calculated by adding these radiative components at first. Only except the three reconstructed products and NOCS V2.0 that providing *R*_*ns*_ and *R*_*nl*_, all of the other products providing four radiative components (*R*_*si*_, *R*_*so*_, *R*_*li*_, and *R*_*lo*_). After that, the ocean surface *R*_*n*_ from all the 11 products were pre-processed into daily means in UTC format, and the six products used for fusion were resampled into six scales (0.05°, 0.1°, 0.25°, 0.5°, 1°, and 2°) using bilinear interpolation^58,59^. Specifically, for fusion modeling, these six resampled products were extracted according to the locations of all moored buoys one by one; for production, the areas of the ocean of these products were first identified by the ocean-land mask originally developed at Boston University based on MODIS data^60^ before resampling. To obtain more reasonable values of *R*_*n*_ over coastal seas, the pixels of these six products along land-sea boundaries were further processed by the creeping sea fill (CSF) method^61^, using which the targeted points were filled through the weight of the eight nearest pixels: a weight of one at the four diagonal points and a weight of two at up, down, left, and right points that have values, the target point is filled if the weights sum to at least three. The CSF method has been widely used for coastline processing^2,62,63^ to reduce the influence of contaminations from land.

### Workflow of data generation and evaluation

**Fig. 2:**
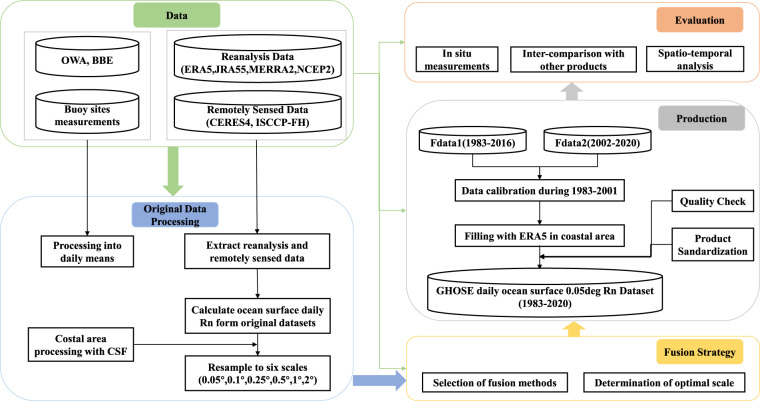
The flowchart of generating and evaluating the GHOSE daily ocean surface *R*_*n*_.

Figure 2 shows a flowchart of the generation and evaluation of the GHOSE daily ocean surface *R*_*n*_. It includes three parts: the development of a fusion strategy, the generation and evaluation of the GHOSE daily ocean surface *R*_*n*_. The fusion strategy involved determining the fusion methods, products of fusion, and the optimal spatial scale based on the *in situ* ocean surface *R*_*n*_ samples from 1988 to 2016, followed by the application of the fusion strategy to generate the GHOSE daily ocean surface *R*_*n*_ from 1983 to 2020. And the evaluation includes direct validation and inter-comparison with other products in accuracy and spatio-temporal variations. The details are provided below.

### Fusion model development

In order to generate a long term ocean surface *R*_*n*_ dataset covering 1983–2020, the fusion was decided to conduct for two periods (1988–2016, 2002–2016) separately because of the different lasting years of the products for fusion (see Table 2) after multiple experiments. Four commonly used data fusion methods, SR, STS, BMA and BP, were explored one by one at six spatial scales (0.05°, 0.1°, 0.25°, 0.5°, 1°, and 2°). ISCCP-FH and the four reanalysis products were used for the fusion model building for 1988–2016 (called Fmod1), CERES4 and the four reanalysis products were used for establishing the fusion model for 2002–2016 (called Fmod2). Note that the beginning year was set to 2002 in Fmod2 because of the unstable performance in CERES4 in 2000–2001^64^. To determine the optimal fusion method for the ocean surface *R*_*n*_, five-fold cross-validation (5-CV) was applied in this study. All training samples were randomly divided into five subsets, of which one subset was used for validation and the remaining four subsets for model fitting. This procedure was repeated five times until each of the five subset had been used for validation and training once, and the statistics of the results of validation were then compiled^65^ and taken as the final results. Three statistical measures were used to represent the accuracy of validation: the root mean-squared difference (RMSD, Wm^−2^), correlation coefficient squared (R^2^), and bias (Wm^−2^).

**Fig. 3:**
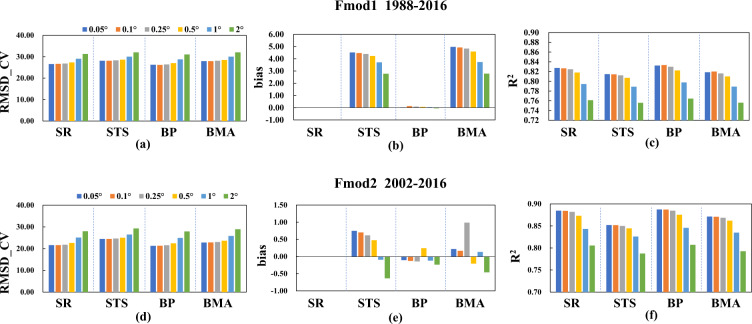
Comparison of the CV accuracy results represented by RMSD (Wm^−2^), bias (Wm^−2^) and R^2^ of four fusion methods (SR, STS, BP, and BMA) at six scales (0.05°, 0.1°, 0.25°, 0.5°, 1°, and 2°) in (**a**–**c**) Fmod1 during 1988–2016 and (**d**–**f**) Fmod2 during 2002–2016.

The results of 5-CV modeling for the four methods at the six spatial scales in Fmod1 and Fmod2 are presented and compared in Fig. 3. The results indicated that the performance of SR and BP was very similar and relatively better than other two methods, with much smaller biases and comparable values of RMSD and R^2^. Combined with the performance of the models at the six spatial scales and their implementation, the SR method at a spatial scale of 0.05° was determined to be the most appropriate fusion strategy, both for Fmod1 and Fmod2, because of its high efficiency and accuracy (RMSDs of 26.6 and 21.57 Wm^−2^, biases of 0.67 × 10^−3^ and –0. 132 × 10^−2^ Wm^−2^, and R^2^ values of 0.83 and 0.88, respectively). The overall performance of Fmod2 was superior to that of Fmod1 for all four fusion methods, with an overall smaller RMSD and larger R^2^. We think that the contribution of CERES4, considered to be one of the most reliable radiative products, was critical here.

Table 3 gives the coefficients of the SR model in the finally determined Fmod1 and Fmod2. The two remotely sensed products (ISCCP-FH and CERES4) occupied a large portion, with larger values in the regression coefficient than other products in Fmod1 and Fmod2. This meant that the performance of the two remotely sensed products was closer to the *in situ* ocean surface *R*_*n*_ than the other four reanalysis products.

**Table 3:** The regression coefficients of the two fusion models.

Regression Coefficients						
Intercept	ISCCP-FH	CERES4	ERA5	JRA55	MERRA2	NCEP R2
**Fmod1 (1988**–**2016, No. of training samples: 45,629)**						
4.936	0.729*	\	0.137*	0.029*	0.017*	0.016*
**Fmod2 (2002**–**2016, No. of training samples: 38,034)**						
−1.302	\	1.001*	0.0235*	0.008*	−0.014*	−0.024*

### Data consistency processing

**Fig. 4:**
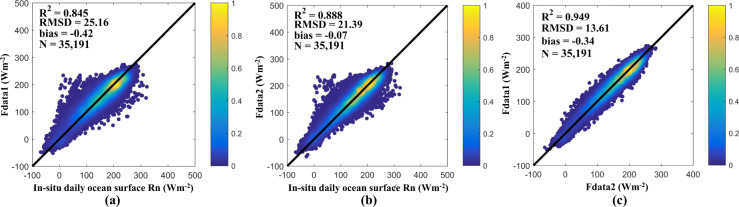
The 5-CV results against the *in-situ* data of (**a**) Fdata1 and (**b**) Fdata2 during 2002–2016, and (**c**) is the scatter plot between Fdata1 and Fdata2 during 2002–2016.

As described above, two fused datasets could be obtained by using the two fusion models namely Fdata1 for 1983–2016 and Fdata2 for 2002–2016 respectively. Hence, to obtain a consistency long-term ocean surface *R*_*n*_, it was necessary to reasonably combine the Fdata1 and Fdata2. The performance of the two fused datasets during the overlapping period of 2002−2016 was examined, and is shown in Fig. 4.

The results showed that Fdata2 was more accurate than Fdata1, with a smaller RMSD of 21.39 Wm^−2^, a smaller bias of –0.07 Wm^−2^, and a higher R^2^ of 0.888 (Fig. 4a,b), although the two datasets were linearly related to each other well (Fig. 4c). Therefore, a simple calibration method was performed by regressing Fdata1 to Fdata2 as below:

3 $${\boldsymbol{Fdata}}{{\bf{1}}}_{{\boldsymbol{r}}}={\bf{1.0116}}\times {\boldsymbol{Fdata}}1{\boldsymbol{-}}{\bf{1.459}}$$

where Fdata1_r_ represents the calibrated Fdata1, and the two regression coefficients were determined based on all samples during 2002–2016. Then, Eq. (3) was used on Fdata1 in 1983–2001 by assuming that the relationship between the datasets was stable. The calibrated Fdata1 for 1983–2001 was then combined directly with Fdata2 from 2002–2016, while the Fdata2 from 2016 onward (2017–2020) could be also combined directly. Based on this, the dataset of a long-term, fused, global daily ocean surface *R*_*n*_ from 1983 to 2020 at 0.05° over global was generated.

### Post-processing

**Fig. 5:**
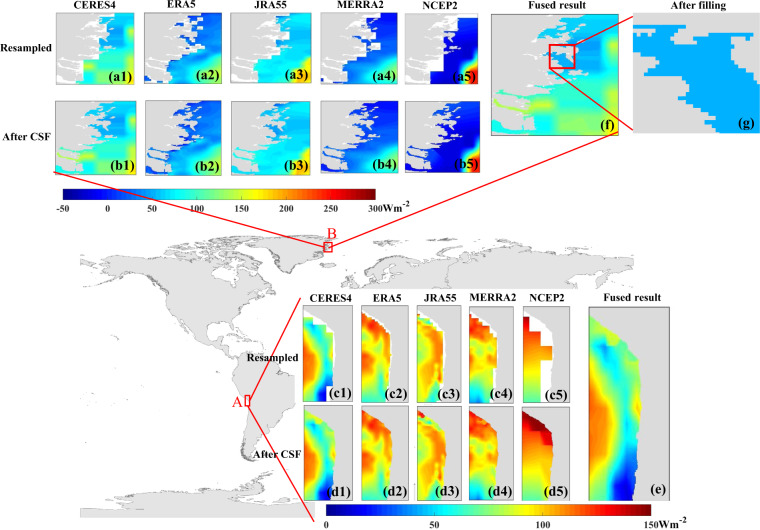
The comparison of two areas with (box B) or without (box A) missing data after fusing: (a1–a5) and (c1–c5) are the daily ocean surface *R*_*n*_ from the five resampled (0.05°) products (CERES4, ERA5, JRA55, MERRA2, and NCEP2) used for fusion, (b1–b5) and (d1–d5) are their corresponding processed results with the CSF method, (**e)** and (**f**) are the fused results, and (**g**) is the final result after filling with the resampled ocean surface *R*_*n*_ from ERA5 within the magnified box in (**f**).

Missing data still persisted after fusion. We use the fused daily ocean surface *R*_*n*_ on a randomly selected day, 150, in 2005 as an example. The details of two areas with (box B) and without (box A) missing data are shown in Fig. 5. The missing data usually appeared over seas very close to coastlines that were irregular, such as the area in box B near Greenland (Fig. 5f), while no missing data were found over coastal seas, where their coastlines were relatively smooth, such as the area in box A in Fig. 5e. For better illustration, the five original products used for fusion, and their processed and resampled results using the CSF method for the two areas are presented in Fig. 5a–d. A comparison of the resampled results of the six products in area A (Fig. 5d1–d5) and area B (Fig. 5b1–b5) suggested that the missing data due to resampling could not be completely filled because of the complicated and irregular coastline, the coarse resolution of the original products, and the limitation of the CSF method itself^61^. All missing data in the final, fused dataset were filled with the resampled daily ocean surface *R*_*n*_ (0.05°) from ERA5 by considering its large spatial resolution and reliable performance. Figure 5g shows the results of filling of the magnified box in Fig. 5f. Finally, the GHOSE daily ocean surface *R*_*n*_ covering the globe at 0.05° from 1983 to 2020 without any missing data was generated.

## Data Records

All GHOSE daily ocean surface *R*_*n*_ from 1983–2020 are freely available from the Big Earth Data Repository (http://data.casearth.cn/)^66^, through which users can link to the specific data entities of each year as well as the user note. The total amount of this dataset is up to 356 Gigabyte. It was stored in thirty-eight folders named after the year, in which each file is stored in the Hierarchical Data File (HDF) format containing the values of daily ocean surface *R*_*n*_ and the corresponding quality check (QC) flags. Daily files are named as “GHOSE07B03.V10.AYYYYDDD.yyyyddd.hdf” where “GHOSE07B03”, “V10”, “YYYY”, and “DDD” denote the product name, version number, year, and Julian day of year (doy), while the lower case “yyyyddd” represents the year and doy of the processing date. All daily values are provided in UTC time in WGS84 geographic coordinate system and stored as 32-bit integer data type in the unit of Wm^−2^ through the conversion by multiplying a scale factor of 0.01, and their valid range was from –150 to 350 Wm^−2^ only with the value of –9999 representing land surface. The QC flags are dimensionless and stored as 8-bit unsigned integer data type for providing information about the sources and quality of the estimates for users reference, and the detailed information is shown in Table 4.

**Table 4:** Explanations of the QC flag in the GHOSE daily ocean surface *R*_*n*_.

Digits	Description	Bit combination	Detailed information
1~2	ocean/land	00	land
		10	ocean
		11	ocean and ice mixing area
3~5	quality	000	missing (no estimated values due to missing input data in some cases)
		001	missing (inland water)
		011	missing (no estimated values caused by interpolation algorithm, filled with neighboring pixel value)
		010	good
		100	accept
		110	uncertainty
6~8	algorithm	000	fusion
		001	filling with CSF before fusion
		011	Filling with ERA5 of the merged vacancy value in coastline area

## Technical Validation

### Direct evaluation

**Fig. 6:**
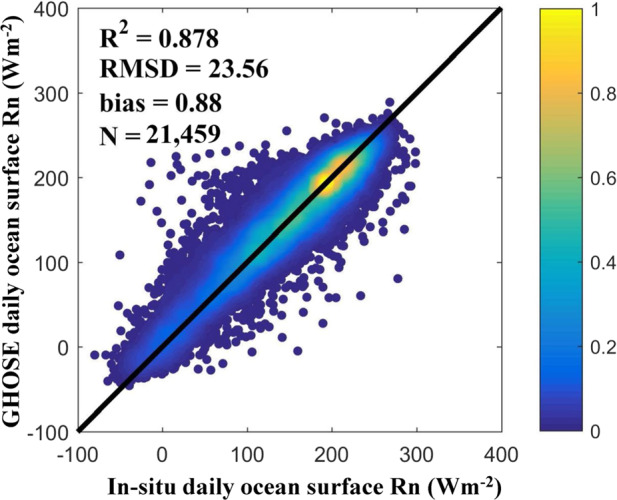
The scatter plot between the daily ocean surface *R*_*n*_ from GHOSE and *in situ* samples during 1988–2019.

A scatter plot between the GHOSE daily ocean surface *R*_*n*_ and the *in situ R*_*n*_ during 1988–2019 is shown in Fig. 6. The overall evaluation of accuracy yielded an R^2^ of 0.878, an RMSD of 23.56 Wm^−2^, and a bias of 0.88 Wm^−2^. As mentioned above, the fusion model was established based on various datasets from 1988 to 2016; hence, the accuracy of evaluation was further examined for the forward period of 2017–2019. Comfortingly, the uncertainties in the estimated ocean surface *R*_*n*_ for the two periods were similar to or even better than those during 1988–2019, with R^2^ values of 0.861 and 0.921, RMSDs of 24.21 and 21.09 Wm^−2^, and biases of 0.37 and 2.72 Wm^−2^, respectively. Hence, the forecasting performance of the fusion model was satisfactory for the short term.

**Fig. 7:**
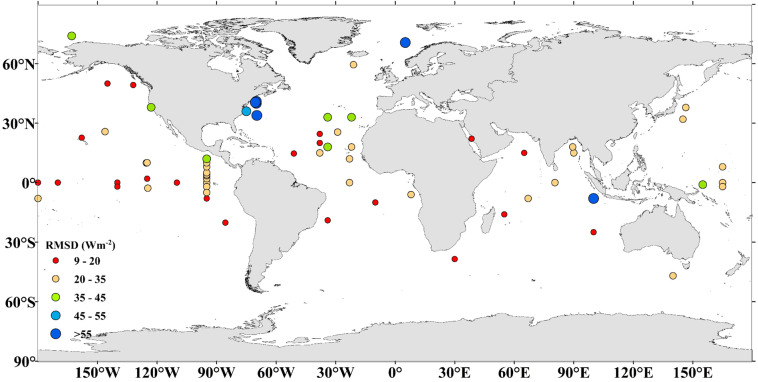
Spatial distribution of RMSD value of the daily ocean surface *R*_*n*_ from GHOSE at 68 moored buoy sites from 1988–2019.

Meanwhile, the spatial distribution of the performance of the GHOSE daily ocean surface *R*_*n*_ represented by the RMSD at each moored site was calculated and presented in Fig. 7. It shows that the closer to the coastal area a moored buoy was, the larger was its estimated uncertainty. Thus, GHOSE should be used with caution in the coastal seas.

And then, the performance of GHOSE was inter-compared with other products. At first, the evaluation accuracy in GHOSE ocean surface *R*_*n*_ and its data sources, the six products used for fusion (CERES4, ISCCP-FH, ERA5, JRA55, MERRA2, and NCEP R2), was calculated against the independent samples during the two fusion periods of 1988–2016 and 2002–2016 and given in Table 5. The daily ocean surface *R*_*n*_ from GHOSE outperformed the original six products for both periods. Specifically, for 1988–2016, the uncertainty of GHOSE was significantly lower than that of ISCCP-FH, with an RMSD of 2.82 Wm^−2^ and a magnitude of bias as large as 9.11 Wm^−2^. For 2002–2016, the uncertainty in GHOSE was the lowest, with an R^2^ of 0.886, an RMSD of 21.88 Wm^−2^, and a bias of 0.35 Wm^−2^, similar to but still better than those of CERES4 — an RMSD lower by 0.27 Wm^−2^ and a bias of nearly 2 Wm^−2^. Combined with the results of evaluation of the six products used for fusion, it seemed that their positive and negative biases could be offset by fusion to obtain a smaller magnitude of the final bias. Moreover, the uncertainty of GHOSE was similar to those in the two remotely sensed products (ISCCP-FH and CERES4) for the two periods. This again demonstrated the significant contribution of the two products to fusion.

**Table 5:** The evaluation results of the daily ocean surface *R*_*n*_ from GHOSE and the six products used for fusing.

product	1988–2016			2002–2016		
	RMSD (Wm^−2^)	bias (Wm^−2^)	R^2^	RMSD (Wm^−2^)	bias (Wm^−2^)	R^2^
CERES4	\	\	\	22.15	2.34	0.88
ISCCP-FH	29.27	9.22	0.824	\	\	\
ERA5	34.73	−0.42	0.712	33.56	0.12	0.726
JRA55	44.53	−5.95	0.562	44.07	−6.06	0.566
MERRA2	43.19	−5.94	0.605	42.41	−6.06	0.613
NCEP R2	50.19	−9.55	0.479	54.04	−8.41	0.401
GHOSE	24.12	0.37	0.861	21.88	0.35	0.886

Furthermore, four other products (GEWEX-SRB, J-OFURO3, OAFlux, and TropFlux) were also evaluated for reference (Table 6). By considering the different durations of the four products, GHOSE was evaluated according to each of their periods for better comparison. The results show that GHOSE outperformed each of the four products. Although the evaluation periods were different, GEWEX-SRB delivered the worst performance in terms of the daily ocean surface *R*_*n*_, with an RMSD of 42.74 Wm^−2^ and a bias of 14.58 Wm^−2^. GHOSE reduced the RMSD and bias by 12.91 and 14.04 Wm^−2^, respectively. It also outperformed OAFlux and TropFlux, reconstructed from ISCCP-FD and ERA-Interim, respectively, in terms of daily ocean surface *R*_*n*_ by recording RMSD values lower by 12.36 and 14.86 Wm^−2^, and the magnitudes of bias lower by 7.71 and 2.32 Wm^−2^, respectively. Among the four products, J-OFURO3 delivered the best performance, slightly worse than that of GHOSE, during its period. They had RMSDs of 24.81 and 24.36 Wm^−2^ and biases of 0.43 and –0.63 Wm^−2^, respectively. This was coincident with the conclusion reported by Chen *et al*.^13^.

**Table 6:** The evaluation results of GHOSE and four reference products in daily ocean surface *R*_*n*_.

product	period	No. of samples	RMSD (Wm^−2^)	bias (Wm^−2^)	R^2^	GHOSE		
						RMSD (Wm^−2^)	bias (Wm^−2^)	R^2^
GEWEX-SRB	1988–2007	4,630	42.74	14.58	0.66	29.83	0.54	0.79
J-OFURO3	1988–2013	12,718	24.81	0.43	0.86	24.36	−0.63	0.86
OAFlux	1988–2009	6,969	36.82	7.72	0.71	24.46	−0.10	0.83
TropFlux	1988–2016	13,557	38.21	−3.28	0.57	23.35	0.96	0.81

### Further comparison with J-OFURO3

**Fig. 8:**
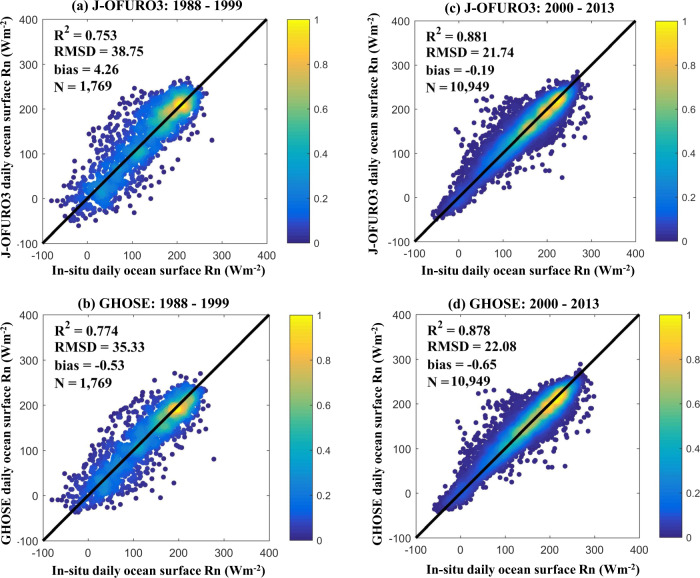
The evaluation results of GHOSE and J-OFURO3 in daily ocean surface *R*_*n*_ against the *in situ* samples during 1988–1999 ((**a**) and (**b**)) and 2000–2013 ((**c**) and (**d**)), respectively.

According to Chen *et al*.^13^, it is worth considering inconsistencies in the daily ocean surface *R*_*n*_ from J-OFURO3 around 2000, which was primarily caused by the change in input datasets from ISCCP-FD to CERES3. We compared GHOSE and J-OFURO3 in terms of the ocean surface *R*_*n*_ for 1988–1999 and 2000–2013 based on the independent evaluation samples. The results are shown in Fig. 8.

**Fig. 9:**
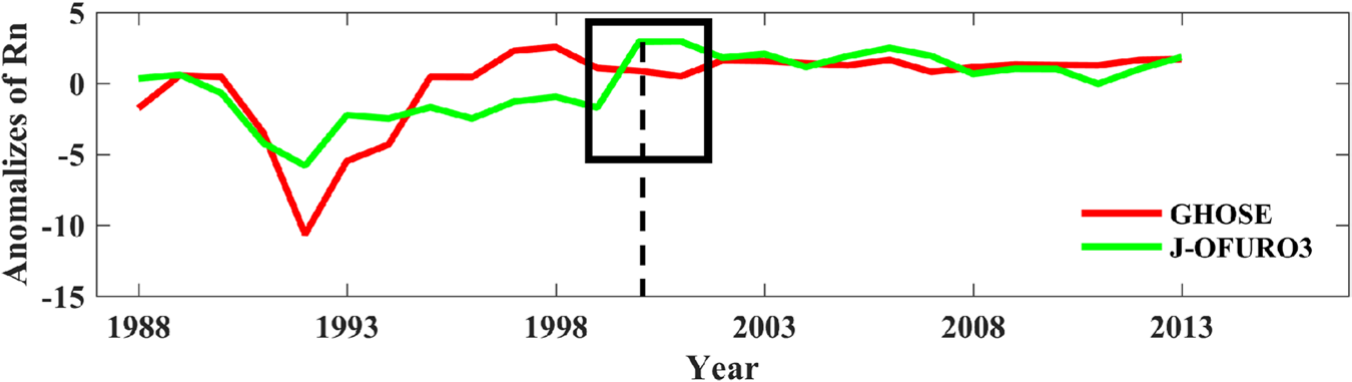
The variations in the anomalies of the global averaged annual mean daily ocean surface *R*_*n*_ from GHOSE and J-OFURO3 during 1988–2013 over the ice-free oceans determined by J-OFURO3. The dashed line indicates the year of 2000.

The evaluation results of both J-OFURO3 and GHOSE were worse before 2000 than after, with RMSD values of 38.75 and 35.33Wm^−2^, respectively, before 2000 (Fig. 8a,b), and 21.74 and 22.58 Wm^−2^ after 2000 (Fig. 8c,d), respectively. The quality of the radiative products was generally poor before 2000, possibly because of the limited available moored buoy measurements (No. of samples = 1,769) in this period. Moreover, a lower uncertainty in the GHOSE daily ocean surface *R*_*n*_ during 1988–1999 occurred possibly because ISCCP-FH was used as the major input in this period. Figure 9 presents the variations in the anomalies of the global averaged annual mean daily ocean surface *R*_*n*_ from GHOSE (red) and J-OFURO3 (green line), and the inconsistency (black box in Fig. 9) for J-OFURO3 was not observed in GHOSE around 2000.

**Fig. 10:**
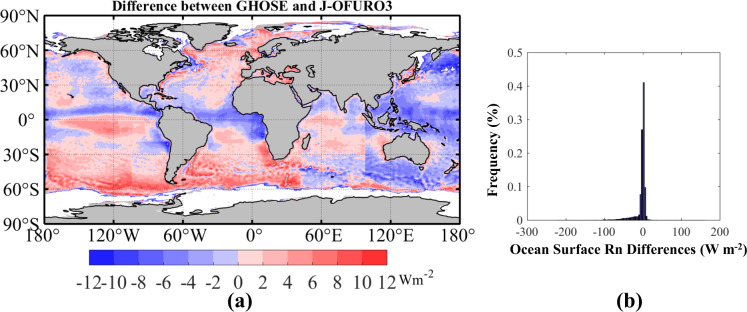
(**a**) The spatial distribution of the differences in the annual mean daily ocean surface *R*_*n*_ by GHOSE minus J-OFURO3 from 1988 to 2013 over global ice-free oceans, and (**b**) its corresponding histogram. White color represents the missing data.

And the spatial distribution and histogram of the differences in the annual mean daily ocean surface *R*_*n*_ obtained by GHOSE minus that of J-OFURO3 during 1988–2013 over global ice-free ocean are shown in Fig. 10a,b. On the whole, the differences in the ocean surface *R*_*n*_ of the two products were mostly at around ± 10 Wm^−2^, but that of GHOSE was mostly smaller than that of J-OFURO3, especially over seas at high latitudes, while the opposite case was obtain over the West Pacific.

**Fig. 11:**
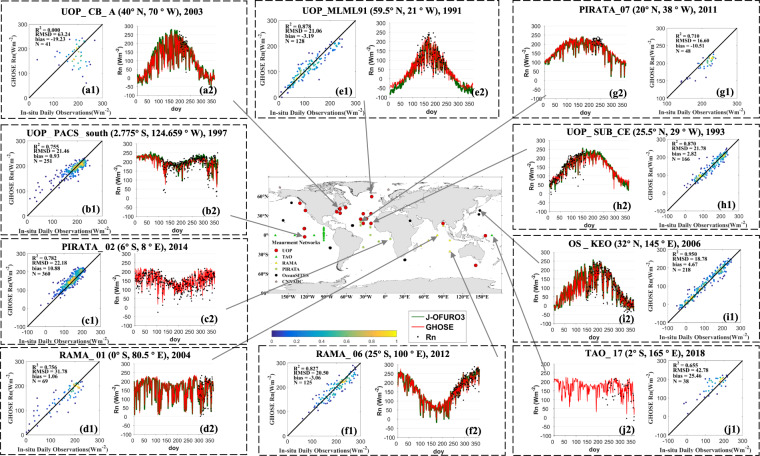
The temporal variations in daily ocean surface *R*_*n*_ from J-OFURO3 (green line), GHOSE (red line), and moored buoy (black dot) and the scatter plot between GHOSE and moored *R*_*n*_ for one randomly selected year at ten moored buoy sites: (a1–2) UOP_CB_A (40°N, 70°W) in 2003, (b1-2) UOP_PACS_south (2.775°S, 124.659°W) in 1997, (c1-2) PIRATA_02 (6°S, 8°E) in 2014, (d1-2) RAMA_01 (0°S, 80.5°E) in 2004, (e1-2) UOP_MLML91 (59.5°N, 21°W) in 1991, (f1-2) RAMA_06 (25°S, 100°E) in 2012, (g1-2) PIRATA_07 (20°N, 38°W) in 2011, (h1-2) UOP_SUB_CE (25.5°N, 29°W) in 1993, (i1-2) OS_KEO (32°N, 145°E) in 2006, (j1-2) TAO_17 (2°S, 165°E) in 2018.

Ten examples over global oceans for one year were randomly selected and are shown in Fig. 11. The variations in the daily ocean surface *R*_*n*_ from J-OFURO3, GHOSE, and moored buoys, and the scatter plot between the results of GHOSE and the *in situ* samples are presented for the 10 sites. Note that J-OFURO3 was not shown when the selected year was beyond its period of 1988–2013. For most cases, the daily ocean surface *R*_*n*_ from the two products and the *in situ* samples were in good agreement, especially at sites over open seas at mid-to-low latitudes, such as UOP_PACS_south (Fig. 11b2), RAMA_01 (Fig. 11d2), RAMA_06 (Fig. 11f2), and PIRATA_07 (Fig. 11g2). For the other cases, the two products agreed well, but were not as close over coastal seas, such as site UOP_CB_A (Fig. 11a2)). Moreover, GHOSE performed generally better than J-OFURO3 before 2000, as exemplified by site UOP_SUB_CE (Fig. 11h2), and at high-latitudes, as in case of site UOP_MLML91 (Fig. 11e2). However, the available *in situ* samples were too few to be used for comprehensive evaluation, such as in the winter season and before 2000, which means more research is needed to better quantify the uncertainty in the daily ocean surface *R*_*n*_ obtained by GHOSE, J-OFURO3, and other products.

### Spatio-temporal analysis and comparison

**Fig. 12:**
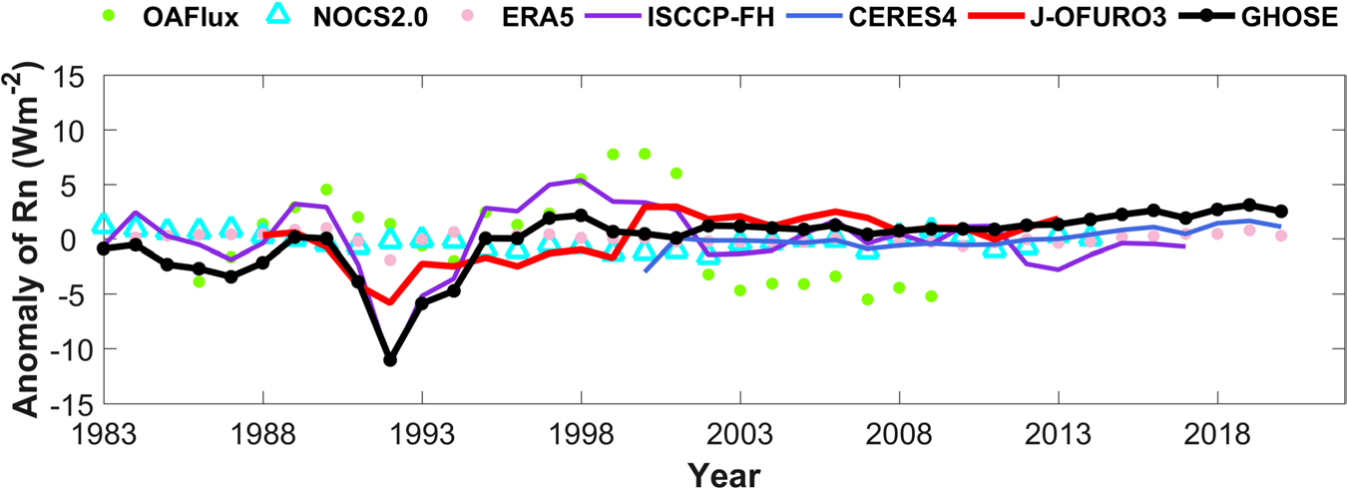
The temporal variations in the global averaged annual mean ocean surface *R*_*n*_ from seven products according to their lasting years during 1983–2020.

Values of the global average annual mean ocean surface *R*_*n*_ from six long term products, including three products used for fusion (ISCCP-FH, CERES4, and EAR5) and three reference products (J-OFURO3, OAFlux, and NOCS2.0), were calculated according to their years of coverage during 1983–2020, and were compared with the results of GHOSE. Note that the area-weighted method, which considers different areas of each pixel by multiplying the cosine of its latitude, was applied to calculate the globally or regionally averaged ocean surface *R*_*n*_. Only ice-free oceans were considered because J-OFURO3 was used. The results are presented in Fig. 12. It shows a large discrepancy among the seven products, especially before 2000. The variations in the ocean surface *R*_*n*_ due to GHOSE were similar to those in ISCCP-FH before 2000 and those of CERES4 afterward. The variations in ERA5 and NOCS2.0 were the smoothest throughout, even around 1992, with a sudden drop caused by the eruption of Mount Pinatubo^67^. This was captured by almost all products, and especially well by NOCS2.0. The variations in J-OFURO3 were still significantly different from those of GHOSE, in addition to the inconsistency around 2000, although the results of their direct evaluation were similar.

**Fig. 13:**
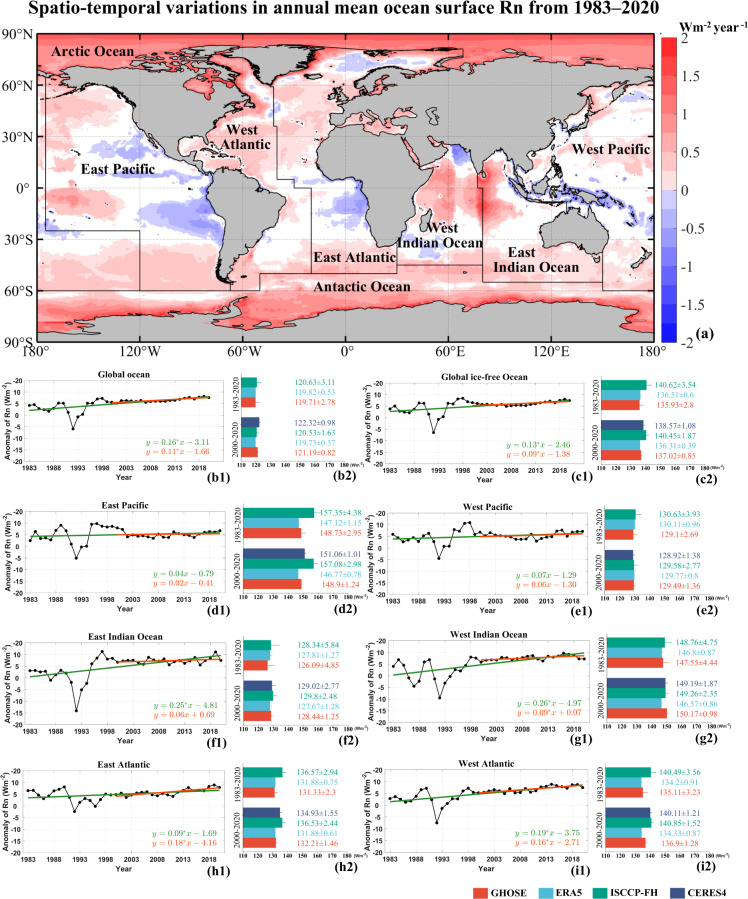
(**a**) The spatial distribution of the long term trend (*p* < 0.05) in the annual mean ocean surface *R*_*n*_ from GHOSE during 1983–2020 over global oceans. (b1)-(i1) are the trends in the anomalies of the area-averaged annual mean daily ocean surface *R*_*n*_ over global oceans, global ice-free oceans and six sub-regions within the global ice-free oceans (55°S–55°N) during 1983–2020 (green line) and 2000–2020 (red line), respectively, and (b2)-(i2) are the area averaged multi-annual mean and standard deviations in ocean surface *R*_*n*_ (Wm^−2^) from four products over the global oceans, global ice-free oceans and the six sub-regions during 1983–2020 and 2000–2020. White color in (**a**) represents the insignificant trend.

The spatial distribution of long term trend (*p* < 0.05) in annual mean GHOSE ocean surface *R*_*n*_ over global oceans during 1983–2020 is calculated using the linear regression method and shown in Fig. 13a. It indicates that most oceans underwent a significant change during 1983–2020 with mostly increasing at mid-high latitudes (red color) and only several increasing (e.g., the Indian Ocean and Tropical Center Pacific) and decreasing hot spots (e.g. the seas to northern Australia, western Africa and South America) over tropical seas, which were coincident with the results of previous studies^13,68–70^. The remarkable increased ocean surface *R*_*n*_ over most oceans at high latitudes where the rate was even as large as 2 Wm^−2^ per year was speculated caused by the increased absorbed solar radiation because of the reduced ocean surface albedo due to the sea ice or glacier melting caused by global warming^71^, but the case was unexpected over the Norway sea (68°N – 80°N, 10°W – 45°E) where the ocean surface *R*_*n*_ overly decreased at a rate of ~0.5 Wm^−2^ per year. According to Kim *et al*.^72^ and Wen *et al*.^73^, the decreased ocean surface *R*_*n*_ occurred possibly because of the decreased SST owing to the inhibited northward flow of Atlantic Meridional Overturning Circulation (AMOC) in this area. This was prompted in turn by the increased freshwater from melting glaciers. In addition, the decrease in *R*_*n*_ over seas near to West Africa might have been caused by biomass burning aerosols (BBAs) over the continent, which cooled the ocean by absorbing shortwave radiation through complex interactions among the ocean, land, and the atmosphere^74,75^. Meanwhile, the long term trends in the area-averaged anomalies of the annual mean GHOSE ocean surface *R*_*n*_ for 1983–2020 and 2000–2020 for global and global ice-free oceans were calculated and given in Fig. 13b1-c1. The global averaged linear trend in Fig. 13b1 over 1983–2020 was in accordance with the results in Fig. 13a, with a significant rate of increase of 0.16 Wm^−2^ per year, which decreased by 0.05 Wm^−2^ per year after 2000, although the positive trend was still significant. Combined with the results in Fig. 13c1, the trend of a relatively smaller increase over global ice-free oceans than that over global oceans further indicated that seas with ice at high latitudes contributed significantly to the increased *R*_*n*_.

For better analysis, the global ice-free oceans (55° S–55° N) were divided into six sub-regions according to the definition provided by the Food and Agriculture Organization of the United Nations (http://www.fao.org/geonetwork, last accessed: 10 Jan. 2020): the East Pacific Ocean, West Pacific, East Atlantic, West Atlantic, East Indian Ocean, and West Indian Ocean, as shown in Fig. 13a. Therefore, the area-averaged linear trends of GHOSE annual mean ocean surface *R*_*n*_ for the two same periods were also calculated for the six sub-regions one by one and given in Fig. 13d1-i1. Based on the results, the ocean surface *R*_*n*_ of nearly all six sub-regions over the global ice-free oceans increased either for 1983–2020 or 2000–2020. Some had a higher rate of increase than the global average (e.g., the Atlantic and Indian Oceans). The ocean surface *R*_*n*_ increased the most quickly and significantly over the Indian Ocean, by more than 0.25 Wm^−2^ per year, during 1983–2020 (Fig. 13f1,g1), especially the West Indian Ocean (Fig. 13g1) at 0.26 Wm^−2^ per year. However, the trend has slowed since 2000 over the entire Indian Ocean, with rates of 0.06 and 0.09 Wm^−2^ per year for the East and West Indian Oceans, respectively. Arora *et al*.^69^ have noted that 70% of the missing heat from the atmosphere is trapped in the Indian Ocean as heat content, which in turn influences the SST. This might explain the increase in *R*_*n*_ in this region. The ocean surface *R*_*n*_ over the West Atlantic also had significant rates of increase of 0.19 Wm^−2^ per year for 1983–2020 and 0.16 Wm^−2^ per year for 2000–2020 (Fig. 13i1). The value over the East Atlantic Ocean increased more slowly, at a rate of 0.09 Wm^−2^ per year over the past 38 years. This rate has been much higher since 2000, at 0.18 Wm^−2^ per year. Only observed in this region, this growth rate is also the highest among the six sub-regions for this period^76^. Over the East and West Pacific (Fig. 13d1,e1), the ocean surface *R*_*n*_ increased but insignificantly, mostly because of the offset of the evenly distributed positive and negative trends in this region, as shown in Fig. 13a. Interestingly, the variations in the ocean surface *R*_*n*_ over the East and West Pacific, where the El Nino Southern Oscillation (ENSO) events occur, also reflected some information on it. For example, the ocean surface *R*_*n*_ increased during two ENSO events of 1987–1988 and 2010–2011 over the East Pacific and vice versa over West Pacific, which should be caused by the variated SST circulation around east-west Pacific^77–79^. Moreover, it seems that the influence of the eruption of Mount Pinatubo on the ocean surface *R*_*n*_ over all global oceans with a sudden drop around 1992.

Afterwards, the area-averaged multi-annual mean and standard deviations in GHOSE ocean surface *R*_*n*_ for global and global ice-free oceans, as well as the six sub-regions were calculated covering 1983–2020 and 2000–2020, respectively. For comparison, the same ones for three products (CERES4, ERA5, and ISCCP-FH) were also calculated. All results are given in Fig. 13b2–i2. Note that the results of CERES4 only for 2000–2020 and that of ISCCP-FH for 1983–2017 and 2000–2017. During 1983–2020, the multi-annual mean ocean surface *R*_*n*_ over global oceans from GHOSE was 119.71 ± 2.78 Wm^−2^, close to that of ERA5 (119.82 ± 0.53 Wm^−2^), and this was also observed for all other regions; while they both were lower than that of ISCCP-FH, especially for the East Pacific (Fig. 13d2) which was thought to be one of the most sensitive regions containing the East Pacific Warm Pool and Cold Tongues with the difference value as large as 10 Wm^−2^. For the period of 2000–2020, the global averaged multi-annual mean ocean surface *R*_*n*_ was 121.19 ± 0.82 Wm^−2^, close to that of ERA5 (119.73 ± 0.37) and a little bit lower than that of CERES4 (122.32 ± 0.98). For the six sub-regions, the multi-annual mean ocean surface *R*_*n*_ from GHOSE was mostly smaller than that of CERES4, except for the West Pacific, and was very close to that of ERA5 for most regions, with a larger value of ~3 Wm^−2^ only over the West Indian Ocean. Similar to the results for 1983–2020, the largest discrepancies in the multi-annual mean ocean surface *R*_*n*_ among the four products were also observed in the East Pacific, followed by the entire Atlantic. Moreover, the standard deviations of the area-averaged multi-annual mean ocean surface *R*_*n*_ from ERA5 were the smallest of the products for all regions considered, which indicates its stability for the ocean surface *R*_*n*_. This is coincident with the results in Fig. 12.

To sum up, the long-term daily ocean surface *R*_*n*_ from GHOSE was satisfactory overall because of robust results, consistent variations, fine spatial resolution, and global ocean coverage without missing data. The results of a preliminary spatio-temporal analysis highlighted its ability to adequately characterize and capture information on variations in the radiative balance on the ocean surface. Therefore, this new dataset offered promise for wide used in the near future.

## Usage Notes

Some abnormal values or mosaics were found in GHOSE ocean surface *R*_*n*_ over some regions for a few days, mainly in 1983 and 1988 (i.e., days 183–196 in 1983, and 153/214–230/ 237–244 in 1988). They occurred primarily because of ISCCP-FH, the quality of the daily ocean surface *R*_*n*_ of which was affected by issues related to the zenith angle of the satellite (https://isccp.giss.nasa.gov/projects/flux.html, last accessed: 8 Jan. 2022) and its *R*_*lo*_. Hence, the daily ocean surface *R*_*n*_ from GHOSE on these days should be used with caution.

## Data Availability

The MATLAB codes for generating and processing data and the CN-NADC *In situ* ocean surface *R*_*n*_ can be accessed at the figshare^80^.
